# The Correlation between High-Sensitivity C-Reactive Protein, Matrix Metallopeptidase 9, and Traditional Chinese Medicine Syndrome in Patients with Hypertension

**DOI:** 10.1155/2013/780937

**Published:** 2013-03-28

**Authors:** Aiming Wu, Dongmei Zhang, Yonghong Gao, Lixia Lou, Haiyan Zhu, Limin Chai, Xiying Lv, Yikun Sun, Lingqun Zhu, Shuoren Wang

**Affiliations:** ^1^Key Laboratory of Chinese Internal Medicine of Ministry of Education, Dongzhimen Hospital Affiliated to Beijing University of Chinese Medicine, Beijing 100700, China; ^2^Key Laboratory of Chinese Internal Medicine of Beijing City, Dongzhimen Hospital Affiliated to Beijing University of Chinese Medicine, Beijing 100700, China; ^3^Department of Cardiology, Dongzhimen Hospital Affiliated to Beijing University of Chinese Medicine, Beijing 100700, China

## Abstract

Hypertension is a common disease affecting millions of people throughout the world. Currently, there is a growing interest in the traditional Chinese medicine (TCM) for patients with hypertension mainly due to the personalized therapy of TCM in many countries. Clinical treatment of patients relies on the successful differentiation of a specific TCM syndrome for hypertension. However, it is difficult to understand that TCM syndrome classifications depend on the clinical experience of a TCM practitioner. Therefore, discovering an objective biomarker associated with TCM syndrome may be beneficial for TCM syndrome classifications. This paper focused on high sensitivity C-reactive protein (HCRP), matrix metallopeptidase 9 (MMP9), and TCM syndrome, and aimed to investigate the relationships between TCM syndrome and the two inflammatory biomarkers in patients with essential hypertension. The result showed that both HCRP and MMP9 are positively correlated with syndrome of wind and phlegm turbidity. Detection of the serum levels of HCRP and MMP9 is beneficial for TCM syndrome classification and prediction of cardiovascular and cerebrovascular risk events in hypertensive patients.

## 1. Introduction

Hypertension is one of the common diseases endangering human health [1, 2]. Numerous studies show that hypertension is an independent risk factor for cardiovascular and cerebrovascular infarction events [3–5]. It is one of the major causes of morbidity and mortality worldwide [6]. Traditional Chinese medicine (TCM) has played an important role in the treatment of hypertension [7, 8]. There has been a growing interest in TCM for patients with hypertension mainly due to the personalized therapy of TCM in many countries. The basic and clinical studies with TCM syndrome classification are becoming increasingly popular [9–12]. Clinical treatments of patients rely on the successful differentiation of a specific TCM syndrome. However, it is not easy to understand this TCM syndrome classification and therefore it requires the clinical experience of a TCM practitioner. Consequently, determining an objective biomarker associated with TCM syndrome may be beneficial for TCM syndrome classification.

The inflammation and TCM syndrome have some interesting links in the context of a disease. Chen et al. explored the relationship between TCM syndromes and inflammatory cytokines in patients with congestive heart failure and found that serum inflammatory cytokines could be taken as the microcosmic indexes for differentiation of the Xin-qi deficiency and Xin-yang deficiency syndrome [13]. Cao et al. confirmed that inflammatory biomarkers are the important markers for microcosmic syndrome differentiation of cold-phlegm syndrome and heat-phlegm syndrome in patients with bronchial asthma [14]. However, the relationship between TCM syndrome and inflammation in hypertension is rarely studied. High-sensitivity C-reactive protein (HCRP) and matrix metalloproteinase-9 (MMP9) are closely associated with inflammation and are now believed to represent the underlying mechanisms leading to the formation of human atheroma. Both proteins are involved in the destabilization of vulnerable plaques and the formation of occlusive thrombus especially in patients with hypertension. TCM syndrome is the integrative response of the intrinsic pathological mechanisms, in the process of disease development. Therefore change of HCRP and MMP9 levels could lead to the change of the TCM syndrome. In other words, HCRP and MMP9 may be one of the biological bases of TCM syndrome.

An earlier study showed that the high levels of serum HCRP and MMP9 are closely associated with the occurrence of stroke and myocardial infarction in hypertensive patients [15, 16]. In addition, changes of the two inflammatory markers have caused changes of TCM syndrome [17–19]. The current study focuses on HCRP, MMP9, and TCM syndrome in an attempt to determine the relationship between TCM syndrome and inflammatory biomarkers in patients with essential hypertension. 

High-sensitivity C-reactive protein (HCRP), the first acute phase protein detected by highly sensitive methods, is a sensitive marker of inflammation [20, 21]. The modest changes in serum HCRP levels can be extremely useful in predicting cardiovascular and cerebrovascular hazardous events in essential hypertensive patients [22–24]. This protein, HCRP, has been a novel risk factor for ischemic stroke, coronary ischemic disease, and overall mortality. In short HCRP is not only an important inflammatory marker but also a direct pathogenic factor of cardiovascular and cerebrovascular diseases [25–28]. In addition to HCRP, MMP9 is another risk factor correlated to atherosclerosis, myocardial infarction, and ischemic stroke. The matrix metalloproteinases (MMPs) are a large family of more than 20 zinc-dependent, extracellularly acting endopeptidases, the substrates of which are proteins of the extracellular matrix and adhesion proteins [29]. Dysregulation of MMP9, a member of the MMP family, may contribute to vascular remodeling during the development and complication of human atherosclerotic lesions. High levels of MMP9 may promote destabilization and complication of atherosclerotic plaques [30]. It is now believed that HCRP and MMP9 represent the underlying mechanisms leading to the formation of human atheroma, favouring both the destabilization of vulnerable plaques and the formation of occlusive thrombus especially in patients with hypertension.

Traditional Chinese medicine (TCM) believes that syndrome can be considered as a reflection of the pathomechanism and physiological mechanism at a certain stage in the development of the disease processing. To some extent, HCRP and MMP9 may reflect both pathological mechanism and the changing of TCM syndrome in hypertensive patients. This study aimed to explore the correlation between HCRP, MMP9, and TCM syndrome and to analyze the possibility of using HCRP and MMP9 to help TCM syndrome classification in hypertension. To achieve this, the serum levels of HCRP and MMP9 were investigated in 268 patients with essential hypertension. The changes of HCRP and MMP9 levels and TCM syndrome in hypertensive subgroups grouped according to the different events of cardiovascular and cerebrovascular infarction were analyzed. The positive rate of TCM syndrome and different levels of HCRP and MMP9 were then analyzed. Differences in serum levels of HCRP and MMP9 between established and nonestablished diagnosis of a TCM syndrome were compared. In addition, the correlation between HCRP, MMP9, and TCM syndrome was investigated. This study indicates that both HCRP and MMP9 are positively correlated with vascular risk events and TCM syndrome and that detection of the serum levels of HCRP and MMP9 may be beneficial for TCM syndrome classification and prediction of cardiovascular and cerebrovascular risk events in hypertensive patients.

## 2. Materials and Methods

### 2.1. Materials

#### 2.1.1. Subjects

From October 2006 to December 2010, a total of 268 subjects were enrolled from Dongzhimen Hospital affiliated to Beijing University of Chinese Medicine (Beijing, China). All selected subjects fulfilled the diagnosis of essential hypertension. Ethical approval was granted by the Ethics Committee of Dongzhimen Hospital affiliated to Beijing University of Chinese Medicine, Beijing, China. Informed consent was obtained by each patient participating in this study.

#### 2.1.2. Diagnostic Standard

The diagnostic standards of essential hypertension referred to the Chinese hypertension clinic guide 2005 (revised edition) [31]. Acute myocardial infarction criteria were found by acute myocardial infarction diagnosis and treatment guideline (Chinese Medical Association) [32]. Cerebral infarction criteria were found by various types of cerebrovascular disease diagnostic points (Chinese Medical Association) [33]. Diagnostic standards of TCM syndromes referred to the following references: (1) criteria of diagnosis and therapeutic effect of diseases and syndromes in traditional Chinese medicine (The People's Republic of China TCM Industry Standard) [34], (2) standard of blood stasis diagnosis (China Association of Integrative Medicine Professional Committee of Huoxiehuayu) [35], (3) standard of TCM syndrome differentiation of deficiency (China Association of Integrative Medicine Professional Committee of Deficiency and Geriatric) [36], (4) TCM diagnosis of hypertension syndrome type standard (Science Press, Beijing, China) [37].

#### 2.1.3. Inclusion Criteria

The inclusion criteria were as follows: (1) essential hypertension, (2) each patient was greater than 35 years of age, and (3) signed the informed consent.

#### 2.1.4. Exclusion Criteria

The exclusion criteria were composed of six conditions: (1) secondary hypertension, (2) acute trauma and surgery, (3) cancer and immune system diseases, (4) pregnant or lactating women, (5) serious diseases of liver, kidney, and hematopoietic system, and (6) patients with allergies or psychosis.

### 2.2. Collection of TCM Clinical Information

Signs and symptoms by four TCM diagnostic methods were collected. These symptoms included the following: hypodynamia, short breath, cardiopalmus, chest pain, chest distress, fainting feeling, amnesia, insomnia, dizziness, irritable tantrum, dryness of mouth, burning sensation of five centers, sighing, depression, heavy limbs, dryness of eyes, tinnitus, sore waist and knee, residual urine, heel pain, sexual dysfunction or infertility, cough, white phlegm, susceptibility to colds, tastelessness in the mouth, bitter taste in the mouth, anorexia, abdominal distension, epigastric fullness, belching, fixity pain or cramps, lower abdominal tenderness, emaciation, menstrual disorders, pachylosis, limb numbness, hypochondrium distending pain, darkish complexion, red complexion, dark color around eyes, dark red lip gingival, pale lips and finger nails, cold abdomen and waist, facial and limb edema, spontaneous perspiration, night sweat, constipation, diarrhea, clear urine in large amounts, yellow urine and oliguria, frequency of micturition at night, swollen tongue body, tooth-marked tongue, thick tongue coating, greasy tongue coating, thick and greasy tongue coating, yellow tongue coating, glossal petechia, lavender subglossal collateral vessels, subglossal collateral vessels engorgement, deep pulse, thready pulse, uneven pulse, and weak pulse. All of the previous TCM clinical information was collected using a unified questionnaire.

### 2.3. HCRP and MMP9 Detection

Patients donated fasting venous blood (4 mL) in the morning. And serum was separated by centrifugation. Serum HCRP levels were measured using Latex-enhanced turbidimetric immunoassay kit (Chemclin Biotech Co., Ltd, Beijing, China) by Beckman CX4 Proautomatic biochemical analyzer. The remaining serum was stored frozen at −20 degrees celsius for MMP9 levels test. Serum MMP9 levels were measured using Human MMP9 ELISA kit (interassay CV 15% with intraassay CV 5%) by CliniBio 128c microplate reader. The HCRP normal reference value is “<3 mg/L” with “≥3 mg/L” regarded as abnormal value. The MMP9 normal reference value is “<140 ng/mL” and its abnormal value is “≥140 ng/mL”. HCRP detection should be performed within 24 hours and MMP9 detection should be detected within three months since the patients were admitted to hospital.

### 2.4. Statistical Analysis

Data were analyzed using SPSS for windows Statistical Package for Social Sciences (version 13.0). The Gaussian distribution and variance homogeneity test were carried out on the measurement data at first. Gaussian variables are expressed as mean ± standard deviation (SD). Independent sample Student's *t* test was carried out between the two groups. Non-Gaussian variables are represented by Median (interquartile range). Differences between the groups were analyzed by using nonparametric tests. Count data are expressed as frequency (percentage). Chi-square test was used to compare the sample rate among the different groups. The Spearman's rank correlation test was performed with analysis of the correlation between HCRP, MMP9, and TCM syndrome. Values of *P* < 0.05 were considered significant.

## 3. Results

### 3.1. The Demographic Information of the Subjects

 A total of 268 (141 males and 127 females) cases of subjects, aged more than 35 years, were included in this study. This group of subjects included 48 cases of patients complicated with acute cardiovascular and cerebrovascular infarctions, 38 cases of patients complicated with obsolete cardiovascular and cerebrovascular infarctions, and 182 cases of hypertensive patients who do not have cardiovascular and cerebrovascular infarction events. The demographic data is shown in Table 1.

### 3.2. TCM Syndrome Distribution in Patients with Essential Hypertension

**Figure 1:**
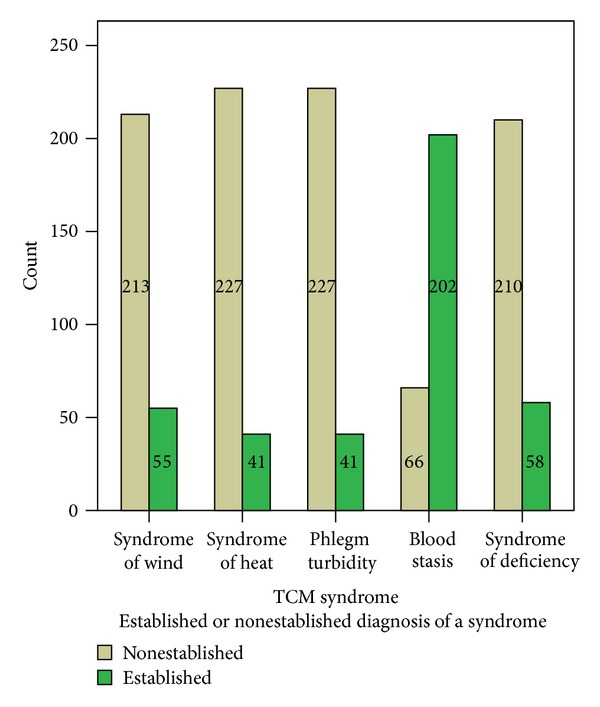
TCM syndrome distribution in patients with essential hypertension.

TCM syndrome classification was confirmed by two TCM senior practitioners with consistent diagnostic opinions. In addition TCM syndrome can be validated by corresponding diagnostic standards of TCM syndromes. TCM syndrome of subjects was divided into the following five main types, including syndrome of wind, heat, phlegm turbidity, blood stasis, and deficiency (Figure 1).

### 3.3. The Positive Rate Comparison of HCRP, MMP9, and TCM Syndrome in Different Hypertensive Subgroups

According to the different events of cardiovascular and cerebrovascular infarctions, 268 cases of essential hypertensive patients were divided into three groups as follows: noncardiovascular and cerebrovascular infarctions group, acute cardiovascular and cerebrovascular infarctions group and obsolete cardiovascular and cerebrovascular infarctions group. Chi-square test was used to compare the positive rate among the different groups. The positive rate of HCRP, MMP9, and TCM syndrome (wind, heat, and deficiency) was shown to be significantly different (*P* < 0.01) (Table 2). 

### 3.4. The Positive Rate Comparison of TCM Syndrome in Different Serum Levels of HCRP and MMP9

According to different serum levels of HCRP, 268 cases of essential hypertensive patients were divided into two groups as follows: “HCRP < 3 mg/L” group and “HCRP ≥ 3 mg/L” group. Similarly these patients were also divided into another two groups as follows: “MMP9 < 140 ng/mL” group and “MMP9 ≥ 140 ng/mL” group. Then Chi-square test was used to compare the positive rate of TCM syndrome between the different groups. The positive rate of wind and phlegm turbidity appeared significantly differently (*P* < 0.01 or *P* < 0.05, resp.) in different serum levels of HCRP and MMP9. Statistical results are shown in Table 3.

### 3.5. The Comparison of Serum Levels of HCRP and MMP9 between Established and Nonestablished Diagnosis of Each TCM Syndrome

**Figure 2:**
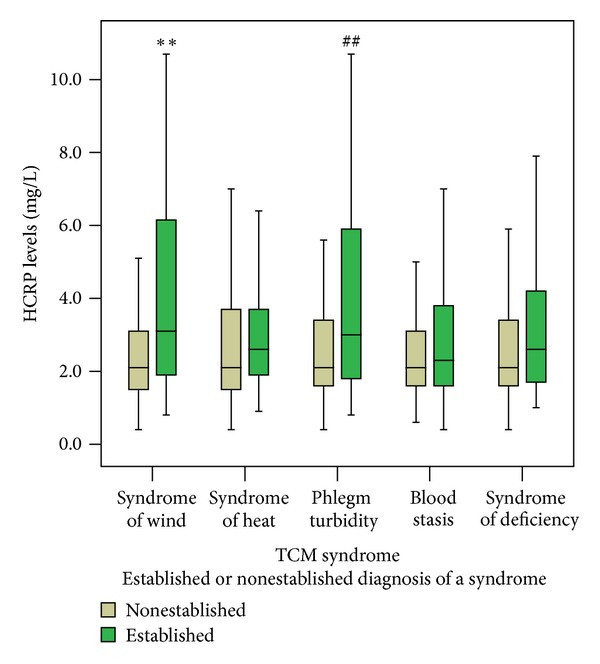
The comparison of serum levels of HCRP between established and nonestablished diagnosis of each TCM syndrome. The serum HCRP levels were compared, respectively, in each TCM syndrome, including syndrome of wind, syndrome of heat, phlegm turbidity, blood stasis, and syndrome of deficiency. Notes: **P < 0.01 versus nonsyndrome of wind, ^##^ *P* < 0.01 versus nonphlegm turbidity.

**Figure 3:**
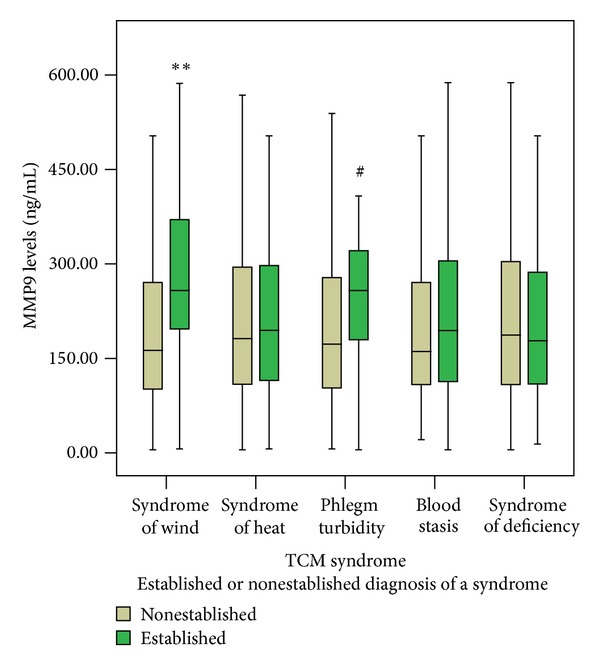
The comparison of serum levels of MMP9 between established and nonestablished diagnosis of each TCM syndrome. The serum MMP9 levels were compared, respectively, in each TCM syndrome, including syndrome of wind, syndrome of heat, phlegm turbidity, blood stasis, and syndrome of deficiency. Notes: **P < 0.01 versus nonsyndrome of wind, ^#^ *P* < 0.05 versus nonphlegm turbidity.

The serum levels of HCRP and MMP9 were compared, respectively, in each TCM syndrome including syndrome of wind, heat, phlegm turbidity, blood stasis, and deficiency. The HCRP and MMP9 levels were not normally distributed and are represented by median interquartile range. There was a significant difference of HCRP levels in syndrome of wind and phlegm turbidity (*P* < 0.01 and *P* < 0.05, resp.) using nonparametric tests (Figure 2). The result of MMP9 levels was the same tendency (Figure 3).

### 3.6. Correlations on Serum Biomarker Levels and TCM Syndrome

Spearman's correlations test was performed to analyze the correlation between HCRP, MMP9, and TCM syndrome. *P*  values < 0.05 were considered significant. The results indicate that there exists some positive correlation between serum biomarker levels and TCM syndrome shown in Table 4. HCRP was positively associated with syndrome of wind (*r* = 0.223, *P* < 0.01) and phlegm turbidity (*r* = 0.162, *P* < 0.01). Matrix metallopeptidase 9 (MMP9) was also positively associated with syndrome of wind (*r* = 0.273, *P* < 0.01) and phlegm turbidity (*r* = 0.152, *P* < 0.05). 

### 3.7. Correlation between HCRP and MMP9

**Figure 4:**
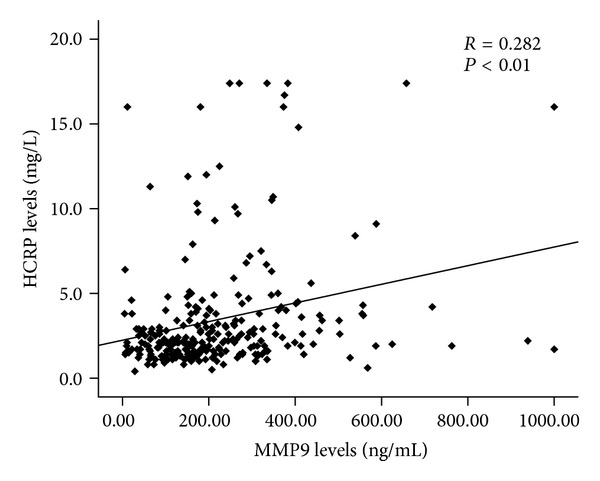
Correlation between HCRP and MMP9.

Correlation between serum HCRP levels and MMP9 levels was analyzed using Spearman's correlations test in patients with hypertension. The results was showed that there is a positive correlation (*r* = 0.282, *P* < 0.01) between HCRP and MMP9 (Figure 4).

## 4. Discussion

Hypertension is one of the common health problems affecting millions of people throughout the world. It can lead to atherosclerotic complications including stroke, coronary heart disease, and heart failure [38, 39]. As some hypertension-related symptoms cannot be completely relieved by western medicine, some patients have turned to TCM, hoping that such treatments might improve their symptoms [40]. Clinical treatments of a patient rely on the successful differentiation of a specific TCM syndrome. TCM syndrome is a reflection of all phenomena of a disease at a certain stage in the development of the disease processing, including etiology, location, pathological mechanisms, and characteristics. To some extent, HCRP and MMP9 may also reflect pathological mechanisms, and numerous studies show that HCRP and MMP9 are now believed to represent the underlying mechanism leading to the formation of human atheroma. In addition both these proteins favour both the destabilization of vulnerable plaques and the formation of occlusive thrombus especially in patients with hypertension [41–47]. It is therefore possible that there may be a kind of relationship yet to be determined, between HCRP, MMP9, and TCM syndrome. This study aimed to determine biomarkers, which may reflect both pathological mechanisms and the changing of TCM syndrome of hypertension, to make TCM syndrome classification more objective and more convenient.

This study indicates that both HCRP and MMP9 are positively correlated with vascular risk events. In terms of TCM syndrome, they are positively correlated with syndrome of wind and phlegm turbidity, and there is a positive correlation between HCRP and MMP9. These results suggest that serum levels of HCRP and MMP9 are a reflection of not only the western pathological but also the TCM pathogenesis performance in patients with essential hypertension. Serum levels of HCRP and MMP9 can indicate progression of disease and changing of TCM syndrome. In addition, they are positively correlated with syndrome of wind and phlegm turbidity. Due to the changeable and accelerated characteristics of wind syndrome, this syndrome often prompts rapid change in the disease. Both HCRP and MMP9 are sensitive biomarkers which may reflect destabilization and complication of atherosclerotic plaques early in the hypertension. It is possible that HCRP and MMP9 are related to syndrome of wind. In addition, according to the understanding of TCM etiology, there is a type of etiology named pathological product etiology. It is not only the product but also a new cause of diseases. Phlegm turbidity and blood stasis are all such TCM etiology. This paper indentifies that both HCRP and MMP9 are positively associated with phlegm turbidity. This may be due to the fact that HCRP and MMP9 are not only important pathological products but also direct pathogenic factors of cardiovascular and cerebrovascular diseases. This study, however, was unable to confirm association between HCRP and MMP9 with blood stasis. The most likely reason for this is that the Standard of Blood Stasis Diagnosis used in this study is too high in sensitivity but without high specificity. The results of the current study indicate that detection of serum levels of HCRP and MMP9 is beneficial for TCM syndrome classification and prediction of cardiovascular and cerebrovascular risk events in hypertensive patients.

The limitations of this study include the sample size. In addition, we did not further refine the classification of TCM syndrome and perform quantitative score. Finally, there is no follow-up observation of the TCM syndrome dynamic evolution. Further research could focus on the solution of the previously mentioned limitations to provide more convincing evidence-based medicine of TCM syndrome.

## 5. Conclusion

This paper focused on HCRP, MMP9, and TCM syndrome and to investigate the relationships between TCM syndrome and the two inflammatory biomarkers in patients with essential hypertension. To achieve this, the characteristics of HCRP, MMP9, and TCM syndrome were compared, and the correlation was analysed in patients with essential hypertension. It was found that the positive rate of HCRP and MMP9 in hypertension complicated by vascular thrombosis event group is higher than that in the nonevent group. The positive rate of syndrome of wind and phlegm turbidity increased in groups with high serum levels of HCRP and MMP9, compared to groups with low serum levels of the two proteins. Furthermore, the levels of HCRP and MMP9 increased in wind syndrome group compared with nonwind syndrome group. In terms of phlegm turbidity syndrome, the tendency is the same. Further analysis showed that both HCRP and MMP9 are positively correlated with syndrome of wind and phlegm turbidity. There is also a positive correlation between HCRP and MMP9. In summary, detection of the serum levels of HCRP and MMP9 is beneficial for TCM syndrome classification and prediction of cardiovascular and cerebrovascular risk events in hypertensive patients.

## Figures and Tables

**Table 1:** The demographic information of the subjects.

Items	Demographic information
Gender	
Males (case (%))	141 (52.6%)
Females (case (%))	127 (47.4%)
Age (years)	62.5 ± 11.1
Duration of hypertension (years)	10.5 ± 10.3
Systolic blood pressure (mmHg)	144.5 ± 21.1
Diastolic blood pressure (mmHg)	81.4 ± 15.1
Essential hypertension without cardiovascular and cerebrovascular infarctions (case (%))	182 (67.9%)
Essential hypertension complicated with acute cardiovascular and cerebrovascular infarctions (case (%))	48 (17.9%)
Essential hypertension complicated with obsolete cardiovascular and cerebrovascular infarctions (case (%))	38 (14.2%)

**Table 2:** The positive rate comparison of HCRP, MMP9, and TCM syndrome in different hypertensive subgroups.

	Cardiovascular and cerebrovascular infarction events				
	No cases (%)	Acute cases (%)	Obsolete cases (%)	*χ* ^2^	*P*
Cases	182	48	38	—	—
Syndrome of wind	27 (14.8%)	23 (47.9%)	5 (13.2%)	26.957	0.000
Syndrome of heat	26 (14.3%)	10 (20.8%)	5 (13.2%)	1.413	0.493
Phlegm turbidity	12 (6.6%)	26 (54.2%)	3 (7.9%)	68.212	0.000
Blood stasis	135 (74.2%)	37 (77.1%)	30 (78.9%)	0.478	0.788
Syndrome of deficiency	45 (24.7%)	2 (4.2%)	11 (28.9%)	10.860	0.004
HCRP ≥ 3 mg/L	45 (24.7%)	31 (64.6%)	12 (31.6%)	27.393	0.000
MMP9 ≥ 140 ng/mL	108 (59.3%)	39 (81.3%)	34 (89.5%)	18.034	0.000

**Table 3:** The positive rate comparison of TCM syndrome in different serum levels of HCRP and MMP9.

	HCRP				MMP9			
	HCRP < 3 mg/L cases (%)	HCRP ≥ 3 mg/L cases (%)	*χ* ^2^	*P*	MMP9 < 140 ng/mL cases (%)	MMP9 ≥ 140 ng/mL cases (%)	*χ* ^2^	*P*
Cases	180	88	—	—	87	181	—	—
Syndrome of wind	25 (13.9%)	30 (34.1%)	14.789	0.000	7 (8%)	48 (26.5%)	12.294	0.000
Syndrome of heat	26 (14.4%)	15 (17.0%)	0.309	0.579	12 (13.8%)	29 (16.0%)	0.225	0.635
Phlegm turbidity	19 (10.6%)	22 (25.0%)	9.517	0.002	7 (8%)	34 (18.8%)	5.229	0.029
Blood stasis	132 (73.3%)	70 (79.5%)	1.229	0.294	62 (71.3%)	140 (77.3%)	1.172	0.279
Syndrome of deficiency	35 (19.4%)	23 (26.1%)	1.561	0.212	18 (20.7%)	40 (22.1%)	0.069	0.793

**Table 4:** Correlations on serum biomarker levels and TCM syndrome.

	Syndrome of wind		Syndrome of heat		Phlegm turbidity		Blood stasis		Syndrome of deficiency	
	*r*	*P*	*r*	*P*	*r*	*P*	*r*	*P*	*r*	*P*
HCRP	0.223	0.000	0.096	0.116	0.162	0.008	0.055	0.367	0.084	0.170
MMP9	0.273	0.000	0.024	0.702	0.152	0.013	0.054	0.381	−0.003	0.960
